# Identification of *Atg2* and *ArfGAP1* as Candidate Genetic Modifiers of the Eye Pigmentation Phenotype of Adaptor Protein-3 (AP-3) Mutants in *Drosophila melanogaster*

**DOI:** 10.1371/journal.pone.0143026

**Published:** 2015-11-13

**Authors:** Imilce A. Rodriguez-Fernandez, Esteban C. Dell’Angelica

**Affiliations:** Department of Human Genetics, David Geffen School of Medicine, University of California Los Angeles, Los Angeles, California, United States of America; CINVESTAV-IPN, MEXICO

## Abstract

The Adaptor Protein (AP)-3 complex is an evolutionary conserved, molecular sorting device that mediates the intracellular trafficking of proteins to lysosomes and related organelles. Genetic defects in AP-3 subunits lead to impaired biogenesis of lysosome-related organelles (LROs) such as mammalian melanosomes and insect eye pigment granules. In this work, we have performed a forward screening for genetic modifiers of AP-3 function in the fruit fly, *Drosophila melanogaster*. Specifically, we have tested collections of large multi-gene deletions–which together covered most of the autosomal chromosomes–to identify chromosomal regions that, when deleted in single copy, enhanced or ameliorated the eye pigmentation phenotype of two independent AP-3 subunit mutants. Fine-mapping led us to define two non-overlapping, relatively small critical regions within fly chromosome 3. The first critical region included the *Atg2* gene, which encodes a conserved protein involved in autophagy. Loss of one functional copy of *Atg2* ameliorated the pigmentation defects of mutants in AP-3 subunits as well as in two other genes previously implicated in LRO biogenesis, namely *Blos1* and *lightoid*, and even increased the eye pigment content of wild-type flies. The second critical region included the *ArfGAP1* gene, which encodes a conserved GTPase-activating protein with specificity towards GTPases of the Arf family. Loss of a single functional copy of the *ArfGAP1* gene ameliorated the pigmentation phenotype of AP-3 mutants but did not to modify the eye pigmentation of wild-type flies or mutants in *Blos1* or *lightoid*. Strikingly, loss of the second functional copy of the gene did not modify the phenotype of AP-3 mutants any further but elicited early lethality in males and abnormal eye morphology when combined with mutations in *Blos1* and *lightoid*, respectively. These results provide genetic evidence for new functional links connecting the machinery for biogenesis of LROs with molecules implicated in autophagy and small GTPase regulation.

## Introduction

The Adaptor Protein (AP)-3 complex is a component of the basic molecular machinery that mediates the trafficking of integral membrane proteins within the endosomal-lysosomal system [1]. It belongs to a family of heterotetrameric complexes, AP-1 through -5 and COPI-F, which originated through gene duplication events that took place before divergence of the last common ancestor of all modern eukaryotes [2]. The AP-3 complex, which is considered the most ancient of the five AP complexes [2], is composed of δ, β3, μ3 and σ3 subunits [1,3]. In mammals, each the β3, μ3 and σ3 subunits is encoded by a pair of paralog genes. Mutations in the gene encoding one of the two variants of the β3 subunit in humans cause Hermansky-Pudlak syndrome (HPS) type 2 [4–11]. Like all other forms of autosomal-recessive HPS, HPS type 2 is characterized by clinical manifestations that originate from defective formation of cell-type-specific, and functionally specialized, lysosome-related organelles (LROs). Thus, all forms of HPS are associated with oculocutaneous albinism and bleeding diathesis owing to abnormal biogenesis of melanosomes and platelet dense granules, respectively [12]. In addition, patients suffering from HPS type 2 are prone to recurrent infections owing to defective LROs in various cells of the innate immune systems [5–11].

Mechanistically, the AP-3 functions as a ‘sorting device’ that, upon recruitment to endosomal membranes from a soluble cytoplasmic pool, recognizes targeting determinants within the cytoplasmic aspect of integral membrane proteins [13]. This molecular interaction results in the incorporation of ‘cargo’ integral membrane proteins into membrane-bounded carriers, which are formed through membrane budding driven by assembly of AP-3 with other cytosolic factors into a protein coat [3,13–15]. The target organelles of this AP-3-dependent trafficking pathway, identified mainly through analyses of AP-3-deficient cells derived from HPS-2 patients and mouse models, turned out to depend on cell type [1,15]. For example, in fibroblasts and other mammalian cell types lacking LROs, AP-3 mediates delivery of integral membrane proteins to lysosomes [4,5,16–19], while in melanocytes it defines a route for the delivery of melanin-synthesizing enzymes to maturing melanosomes [20–22].

Several of the factors that cooperate with AP-3 in the trafficking of integral membrane proteins to lysosomes or LROs have been identified through protein-protein interaction analyses, either targeted towards candidates based on prior biological evidence or unbiased proteomic methods [23–28]. While successful, such approach is intrinsically limited to proteins that interact directly with AP-3 or indirectly through other components of the interaction network. Alternative approaches, such as those based on the analysis of genetic interactions, may uncover functional relationships–within the AP-3-dependent trafficking pathway or with other cellular activities–regardless of the existence or absence of direct physical interactions. However, few non-additive genetic interactions involving the AP-3 complex have been reported in metazoans. In mice, phenotypic enhancement effects were observed for homozygous double mutant mice deficient in AP-3 and each of three protein complexes named Biogenesis of Lysosome-related Organelles Complex (BLOC)-1 through -3 [26,29,30], which like AP-3 are involved in the biogenesis of LROs and associated with various forms of human HPS [12], as well as in double mutant mice deficient in AP-3 and OCA2 [31], with the latter being the product of the gene mutated in oculocutaneous albinism type 2 [32]. Understandably, these genetic interaction studies were limited to targeted approaches owing to the practical limitations of inter-breeding multiple mouse lines. Phenotypic enhancement (possibly additive) effects have also been observed in the worm, *Caenorhabditis elegans* [33–36], and the fruit fly, *Drosophila melanogaster* [37–39], through targeted analyses of double mutants deficient in the AP-3 complex and other factors previously implicated in LRO biogenesis. Additional targeted approaches have led to the observations of synthetic lethality in double-mutant worms lacking AP-3 and the endocytic protein Disabled [40] as well as non-additive phenotypic enhancement effects in AP-3-deficient flies carrying a weak allele of *white* (*w*) [41] or misexpressing the endocytic protein Auxilin [39]. Finally, AP-3 mutant alleles have been identified as genetic modifiers of morphological phenotypes induced by misexpression in flies of Blue Cheese [42], which is a protein involved in selective disposal of ubiquitinated protein aggregates [43], the E3 ubiquitin ligase Deltex [44], and human β-amyloid peptide [45].

In this paper, we systematically searched for genetic modifiers of AP-3 function in the fly eye. Prior to the demonstration that mutations in genes encoding AP-3 subunits cause HPS-2 in humans [4] and related phenotypes in mouse models [17,46,47], the *Drosophila* gene *garnet* (*g*) had been shown to encode the fly counterpart of the δ subunit of AP-3 and to be required for normal biogenesis of eye pigment granules [48,49], which like mammalian melanosomes are LROs [50]. Using a hypomorphic allele of this gene for screening, we have identified distinct chromosomal regions on chromosome 3, containing the genes *Atg2* and *ArfGAP1*, which upon gene dosage reduction ameliorated the eye pigmentation phenotype caused by AP-3 deficiency. To our knowledge, this is the first report of an unbiased, forward screening for genetic modifiers of AP-3 function.

## Materials and Methods

### Fly strains and collections

Flies were raised at 25°C using standard husbandry procedures [51]. The fly line *g* ^*2*^, which was derived by multiple outcrosses of the triple mutant strain *y* ^*1*^ *wy* ^*2*^ *g* ^*2*^ (from the Bloomington Drosophila Stock Center) into the genetic background of Canton-S, was kindly provided by Dr. David E. Krantz (University of California, Los Angeles, CA, USA). The fly strain *ArfGAP1* ^*G3-85*^ (formerly *Gap69C* ^*G3-85*^) was kindly provided by Dr. Vladimir E. Alatortsev (Russian Academy of Sciences, Moscow, Russia) [52]. The mutant fly line *blos1* ^*ex2*^ had been generated in our laboratory [39]. All other fly strains, including Canton-S (used as ‘wild-type’ control), *eyg* ^*1*^, *ltd* ^*1*^, and those of the “classic” Bloomington Deficiency Kit for chromosomes 2 and 3 and the Bloomington Deficiency Kit of molecularly defined deletions for chromosome 4 [53], were obtained from the Bloomington Drosophila Stock Center.

### Fly eye microscopy and imaging

Fly eyes were visualized on live adults anesthetized with CO_2_ using a Zeiss Stemi 2000-C Stereo microscope (Carl Zeiss, Thornwood, NY, USA). Images were acquired using a Nikon Coolpix P5000 digital camera adapted to the same microscope.

### Quantification of eye pigments

Red (pteridines) and brown (ommochromes) pigments were extracted from pooled heads of four (for red pigments) or eight (for brown pigments) male flies at ~3 days after eclosion (range 2–5 days) and quantified as previously described [38]. In each experiment, pigment content values were normalized to those of White-null (*w* ^*1118*^) flies and wild-type (Canton-S) flies, which were quantified in parallel (2–3 biological replicates per experiment) and served as 0% and 100% controls, respectively.

### Statistical analyses

Statistical analyses were performed using GraphPad Prism 5.0b (GraphPad Software, San Diego, CA, USA). Comparisons between more than two groups of data were carried out using one-way ANOVA followed by either Dunnett’s (for comparison of multiple data groups to a single common control) or Bonferroni’s (for comparison of selected pairs of data groups within a multiple comparison analysis) post-hoc tests, as indicated in each figure or table legend.

## Results and Discussion

### Primary and secondary screening for genomic regions carrying modifiers of AP-3 function

**Fig 1:**
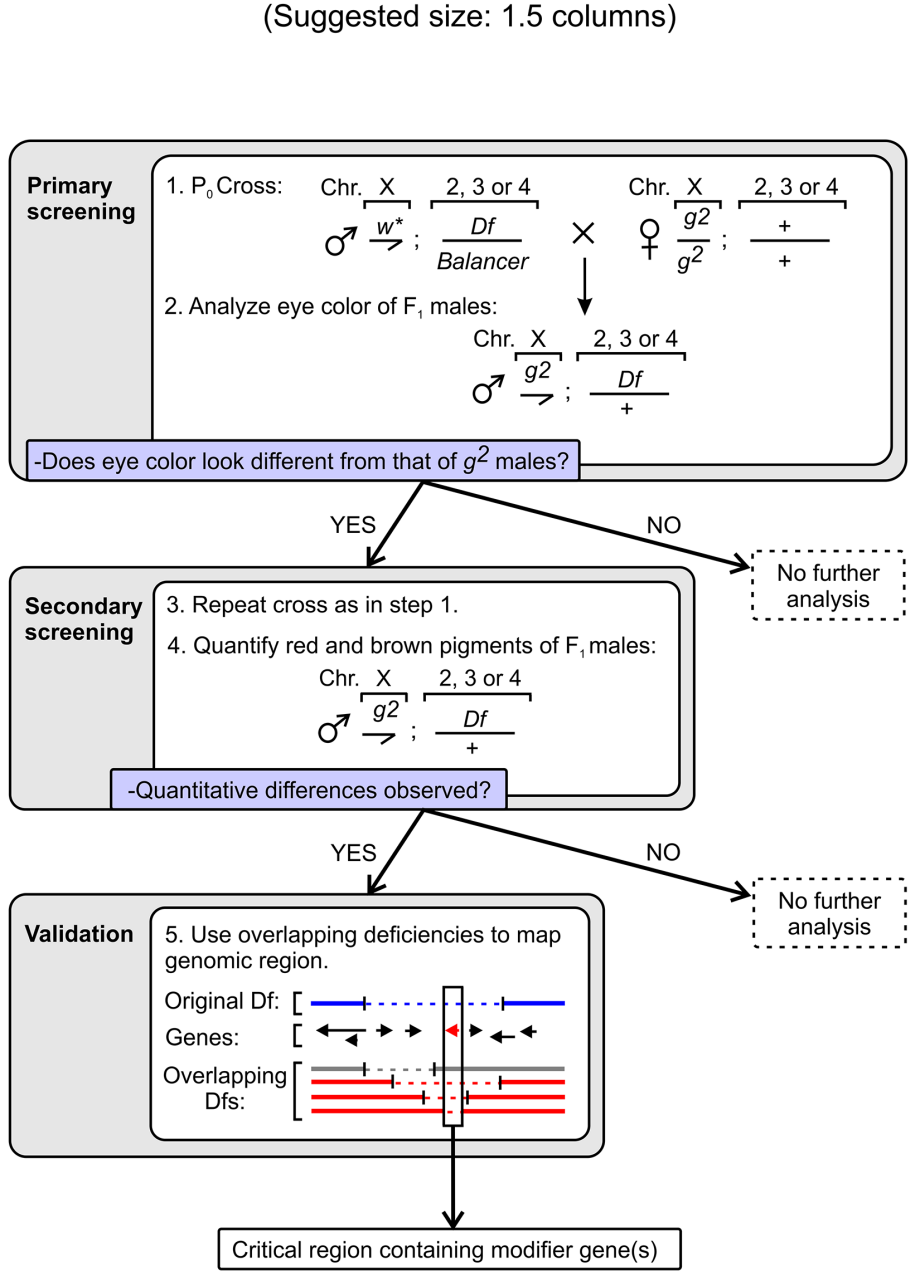
Strategy for the genetic modifier screening carried out in this study. The primary screening involved parental (P_0_) crosses between male flies carrying a deficiency (*Df*) over a balancer in an autosomal chromosome (Chr. 2, 3 or 4) and female flies homozygous for the hypomorphic *g* ^*2*^ allele of the X-linked gene encoding the δ subunit of AP-3. The eye color of male progeny (F_1_) carrying one copy of each deficiency (without the balancer) was compared with that of control *g* ^*2*^ males and, if deemed different, the corresponding deficiency was selected for a secondary screening involving the same P_0_ cross followed by quantification of red and brown pigments in the F_1_ males carrying the deficiency. In cases in which differences in both red and brown pigment content were statistically significant, further validation and fine mapping was attempted using independent deficiency lines in which the deleted genomic regions partially overlapped with that of the deficiency identified through screening. When successful, theses steps allowed identification of a relatively small genomic region (rectangle) containing a modifier gene of interest (red arrow).

In order to identify regions within the autosomal chromosomes of *D*. *melanogaster* (chromosomes 2, 3 and 4) potentially bearing genes that, when in hemizygous form due to deletion of one copy, modify the severity of the phenotype of AP-3-mutant flies, a genetic screening was undertaken following the strategy depicted in Fig 1. In both primary (qualitative) and secondary (quantitative) steps of the screening, male flies carrying large multi-gene deletions (‘deficiencies’) from the so-called ‘Bloomington Deficiency Kit’ collections were crossed with female flies homozygous for the *g* ^*2*^ mutation (on chromosome X) to obtain F_1_ males that were hemizygous for *g* ^*2*^ and heterozygous for a given deficiency over a normal chromosome; for each deficiency, the eye color of these F_1_ males was compared to that of control *g* ^*2*^ males first by light microscopy and, if selected for secondary screening, by pigment extraction and quantification (Fig 1). The *g* ^*2*^ mutation in the gene encoding the δ subunit of AP-3 was chosen not only because of its genomic location on the X chromosome (thus minimizing the number of genetic crosses required to test each deficiency) but also because it represents a weak hypomorph of the *garnet* allelic series [54]. In our hands, the eyes of male adult flies carrying the *g* ^*2*^ allele in the Canton-S genetic background contain 25–30% of the red pigment content of those of the control Canton-S line, whereas those of males carrying one of the strongest alleles of the same gene, *g* ^*53d*^, contain less than 5% of wild-type levels of red pigments (see, for example, Ref. [39]). Thus, the *g* ^*2*^ line was used as a sensitized strain with which both phenotypic enhancers and suppressors could potentially be identified.

**Fig 2:**
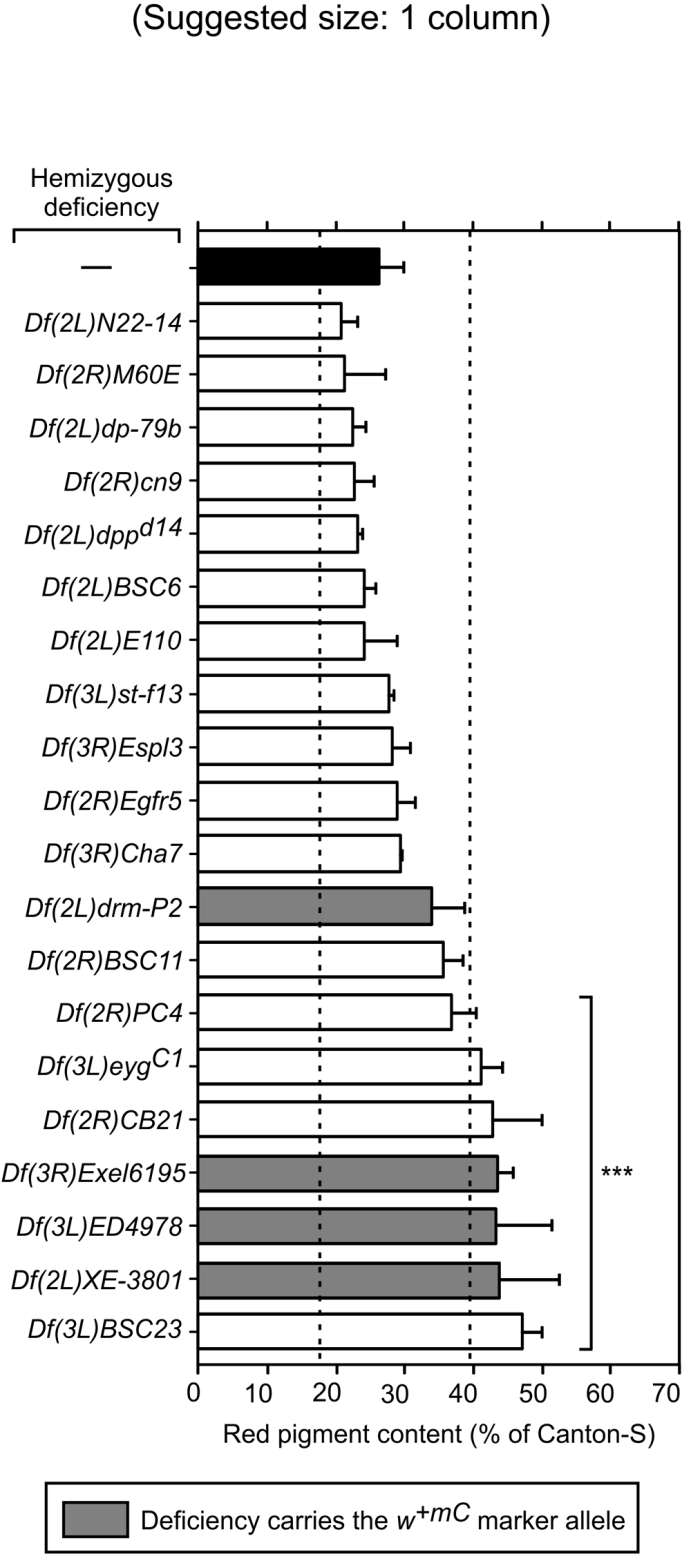
Red pigment content in eyes of *g* ^*2*^ mutant flies carrying hemizygous deletions selected through primary screening. Red pigments were extracted from the heads of adult *g* ^*2*^ mutant males carrying no deletions (—) or a single copy of the indicated deficiencies, quantified as described under Materials and Methods, and expressed as percentages of the red pigment content of male flies of the wild-type line (Canton-S). Bars represent means + SD of 2–28 biological replicates. Grey bars denote values obtained for flies carrying the marker allele *w* ^*+mC*^ linked to the deficiency. Dashed lines indicate threshold values corresponding to 66.7% (2/3) and 150% (3/2) of the pigment content of control *g* ^*2*^ flies carrying no deletion (black bar). One-way ANOVA followed by Dunnett’s test of each group versus *g* ^*2*^ flies carrying no deletion: ***p<0.001.

Out of 213 deficiency lines screened, which together covered >92% of fly autosomal chromosomes 2–4, twenty were selected for secondary screening on the basis of apparent differences in eye color of the corresponding F_1_ males (*g* ^*2*^ hemizygous carrying a copy of the deficiency) relative to *g* ^*2*^ males upon visualization under a dissecting microscope. Of these twenty candidates, seven resulted in statistically significant changes in red pigment levels, upon correction for multiple testing, with all of these changes representing increased pigment content in the eyes of the corresponding F_1_ males relative to those of *g* ^*2*^ controls (Fig 2). It is possible that some of the remaining candidate deficiencies might have modified the eye color phenotype of *g* ^*2*^ mutants through quantitative changes in brown pigment levels only. Nevertheless, given that our goal was to identify modifier genes that might act at the level of pigment granule biogenesis and/or dynamics, thus affecting the accumulation of both pigment types in these granules, those candidate deficiencies that failed to elicit significant changes in red pigment content were not characterized any further. In addition, one of the seven candidates resulting in statistically significant differences, *Df(2R)PC4*, was excluded from further analysis because the relative increase in red pigment content was less than a threshold of 50% increase, which we had arbitrarily set to focus our efforts on the most promising candidates (Fig 2).

The six selected deficiencies were then tested for their ability to modify, when carried in single copy, the brown pigment content of *g* ^*2*^ flies (to verify that both pigment types, not just red pigments, were affected) as well as the red pigment content of *rb* ^*1*^ flies (which carry a hypomorphic mutation in the gene encoding the β3 subunit of AP-3 [55,56]) and wild-type (Canton-S) flies. The results of these experiments are listed in Table 1. Upon correction for multiple testing, the statistical analyses suggested that: (*i*) each of the six deficiencies modified the pigmentation phenotype of an independent AP-3 mutant (*rb* ^*1*^); (*ii*) four of them also altered the red pigment levels of wild-type flies; and (*iii*) all but one, *Df(2R)CD21*, modified the brown pigments levels of *g* ^*2*^ flies (Table 1) in addition to modifying their red pigment content (Fig 2). Together, the primary and secondary screening yielded one genomic region on chromosome 2, which is deleted in deficiency *Df(2L)XE-3801*, and four independent genomic regions on chromosome 3, which are deleted in deficiencies *Df(3L)eyg* ^*C1*^, *Df(3R)Exel6195*, *Df(3L)ED4978*, and *Df(3L)BSC23*, for validation and further characterization.

**Table 1:** Pigment content in flies carrying deficiencies in different genetic backgrounds.

Deficiency	Brown pigment	Red pigment	
	*g* ^*2*^ background	*rb* ^*1*^ background	Canton-S background
—	61.8 ± 2.8 (5)	16.5 ± 1.7 (12)	100 ± 8 (3)
*Df(3L)eyg* ^*C1*^	68.5 ± 3.7 (4)*	33.7 ± 3.4 (6)***	120 ± 8 (5)^NS^
*Df(2R)CB21*	65.0 ± 4.8 (4)^NS^	26.1 ± 2.0 (8)***	121 ± 13 (4)^NS^
*Df(3R)Exel6195*	75.3 ± 1.2 (3) ***	31.0 ± 2.3 (7)***	131 ± 12 (4)**
*Df(3L)ED4978*	74.3 ± 1.2 (3)***	32.0 ± 3.7 (5)***	128 ± 12 (4)*
*Df(2L)XE-3801*	68.8 ± 2.1 (3)*	34.3 ± 3.4 (6)***	123 ± 12 (5)*
*Df(3L)BSC23*	78.3 ± 4.5 (3)***	39.2 ± 0.8 (5)***	132 ± 12 (9)**

Brown or red pigment content in the eyes of adult male flies carrying no deletions (—) or a single copy for each of the indicated deficiencies was measured as described under Materials and Methods and expressed as a percentage of the corresponding pigment content in the eyes of adult male flies of the control line Canton-S. Values represent means ± SD of the numbers of biological replicates indicated between parentheses. One-way ANOVA followed by Dunnett’s test of each group versus the corresponding control carrying no deficiency in the same background: NS, not significant

*p<0.05

**p<0.01

***p<0.001.

### Attempts to validate candidate genomic regions deleted in deficiencies carrying the marker ‘*mini-white*’

**Fig 3:**
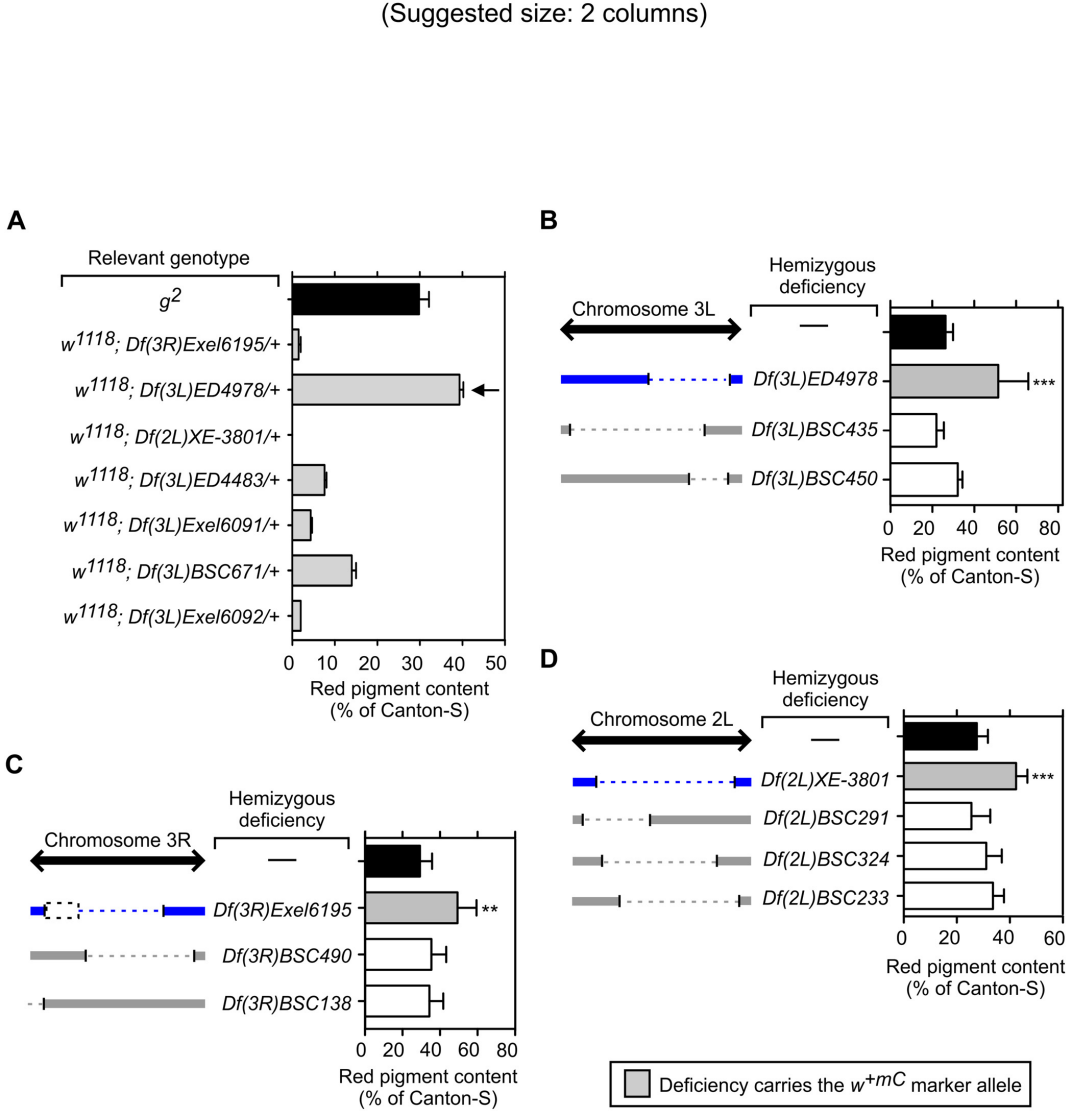
Attempts to validate selected deficiencies carrying the *w* ^*+mC*^ marker as genetic modifiers of the *g* ^*2*^ mutation. (A) Red pigments were extracted from the heads of AP-3-deficient (*g* ^*2*^) or White-negative (*w* ^*1118*^) adult males carrying single copies of the indicated deficiencies with their associated *w* ^*+mC*^ marker. The extracted pigments were quantified as described under Materials and Methods, and the resulting values expressed as percentages of the pigment content of wild-type (Canton-S) flies. Bars represent means + SD of 2–10 biological replicates. Notice that the activity of the *w* ^*+mC*^ marker associated with deficiency *Df(3L)ED4978* resulted in a red pigment content (arrow) higher than that of *g* ^*2*^ flies (black bar). (B-D) Analyses of red pigment content in the eyes of adult *g* ^*2*^ mutant males carrying no deletions (—), single copies of the deficiencies *Df(3L)ED4978* (B), *Df(3R)Exel6195* (C) and *Df(2L)XE-3801* (D) that had been identified through screening, or single copies of deficiencies with partially overlapping deletions and devoid of the *w* ^*+mC*^ marker. Schematic representations of the extent of overlap between the chromosomal regions deleted in the deficiencies identified through screening (blue) and the others (grey) are included in each figure panel. Notice in (C) that a small portion of the chromosomal region deleted in *Df(3R)Exel6195* (dashed box) did not overlap with any of those deleted in other available deficiencies. One-way ANOVA followed by Dunnett’s test of each group versus control *g* ^*2*^ flies carrying no deletion (black bars): **p<0.01; ***p<0.001.

Three of the five deficiencies selected for validation, namely *Df(2L)XE-3801*, *Df(3R)Exel6195*, and *Df(3L)ED4978*, carried the construct *mini-white* (*w* ^*+mC*^) as a genetic marker. It is well known that expression of this construct, which represents a truncated version of the *w* gene lacking some regulatory sequences, varies significantly depending upon the site of chromosomal insertion [57]. Given that high levels of White protein overexpression had been reported to increase, albeit modestly, eye pigmentation of AP-3 δ mutants [41], the possibility of ‘false-positive’ hits in our screening owing to high expression of the *w* ^*+mC*^ construct deserved consideration. To begin to address this issue, genetic crosses were set up to generate male flies hemizygous for the White-null allele *w* ^*1118*^ (on chromosome X) and heterozygous for each of the three deficiencies; in these flies, White function derived exclusively from expression of the *w* ^*+mC*^ construct. As shown in Fig 3A (arrow), the *w* ^*+mC*^ construct carried by one of the three deficiencies, *Df(3L)ED4978*, on a White-null background resulted in red pigment levels of almost 40% of wild-type levels. In further support of the notion that deficiency *Df(3L)ED4978* might have represented a false-positive hit in our screening, none of two ‘overlapping’ deficiencies–with deletions that together covered the entire region deleted in *Df(3L)ED4978* –modified the eye pigmentation phenotype of *g* ^*2*^ flies (Fig 3B). Although the *w* ^*+mC*^ construct carried by the other two deficiencies, *Df(2L)XE-3801* and *Df(3R)Exel6195*, resulted in very little pigmentation on a White-null background (Fig 3A), attempts to validate their phenotypic modifier effect observed on the *g* ^*2*^ background, using independent overlapping deficiencies, were nonetheless unsuccessful (Fig 3C and 3D). It should be noted, however, that the deficiencies available for validation did not completely cover the genomic region deleted in *Df(3R)Exel6195*, thus leaving open the possibility that the partial phenotypic suppression effect elicited by this deficiency could have been caused by hemizygous deletion of a gene in the region that was not deleted in any of the other deficiencies tested (Fig 3C, dashed rectangle). Interestingly, this region turned out to contain a single gene, *CG31145*, which was reported to encode a Golgi-localized protein with casein-kinase activity [58]. Further investigation will be required to test the possibility of *CG31145* being a genetic modifier of AP-3 function in the fly eye.

### Validation and fine-mapping of the genomic regions responsible for the modifier effects elicited by *Df(3L)eyg* ^*C1*^ and *Df(3L)BSC23*

The partial suppressor effects elicited by deficiencies *Df(3L)eyg* ^*C1*^ and *Df(3L)BSC23* on the eye pigmentation phenotype of AP-3-mutant flies could be successfully validated by independent deficiencies with partially overlapping deletions.

**Fig 4:**
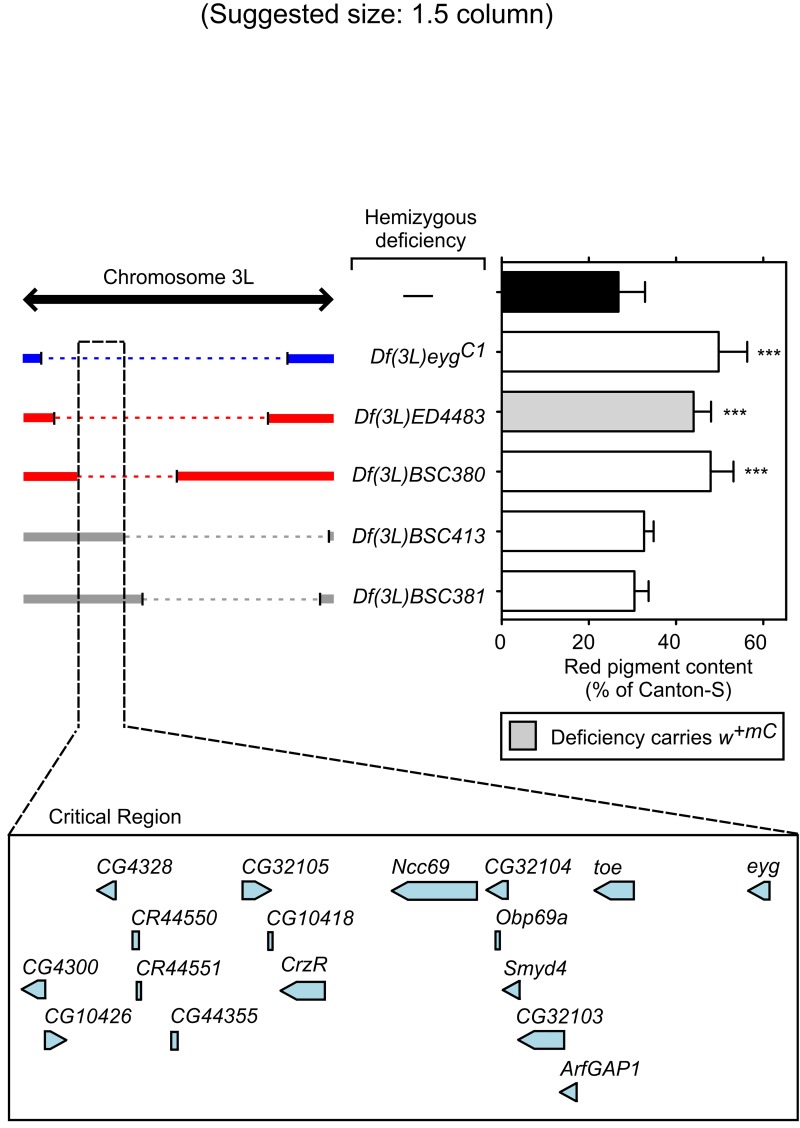
Validation and fine mapping of the critical region responsible for the modifier effect observed for *Df(3L)eyg* ^*C1*^. Red pigments were extracted from the heads of adult *g* ^*2*^ mutant males carrying no deletions (—) or a single copy of the indicated deficiencies, quantified as described under Materials and Methods, and expressed as percentages of the red pigment content of male flies of the wild-type (Canton-S) line. Bars represent means + SD of 6–10 biological replicates. One-way ANOVA followed by Dunnett’s test of each group versus *g* ^*2*^ flies carrying no deletion (black bar): ***p<0.001. Shown on the left is a schematic representation of the extent of overlap between the chromosomal region deleted in the deficiency that had been identified through screening (blue) and those deleted in independent deficiencies that elicited (red) or failed to elicit (grey) a similar modifier effect on the *g* ^*2*^ eye color phenotype. The critical genomic region responsible for the observed modifier effect is highlighted with black dashed lines, and the relative location of genes found within this region (adapted from the FlyBase database) is depicted at the bottom.

In the case of *Df(3L)eyg* ^*C1*^, four independent deficiencies were available to cover the entire genomic region deleted in the original deficiency, and two of them elicited partial suppressor effects on the phenotype of *g* ^*2*^ flies that were akin to that observed for *Df(3L)eyg* ^*C1*^ (Fig 4). Although one of these two deficiencies, *Df(3L)ED4483*, carried the *w* ^*+mC*^ construct as a marker, expression of this construct was deemed low on the basis of the red pigment content of flies carrying such deficiency on a White-null background (Fig 3A). Through comparison of the genomic regions deleted in the deficiencies that elicited the modifier effect and those that failed to do so, a critical region comprising 17 annotated genes was defined (Fig 4, bottom box).

**Fig 5:**
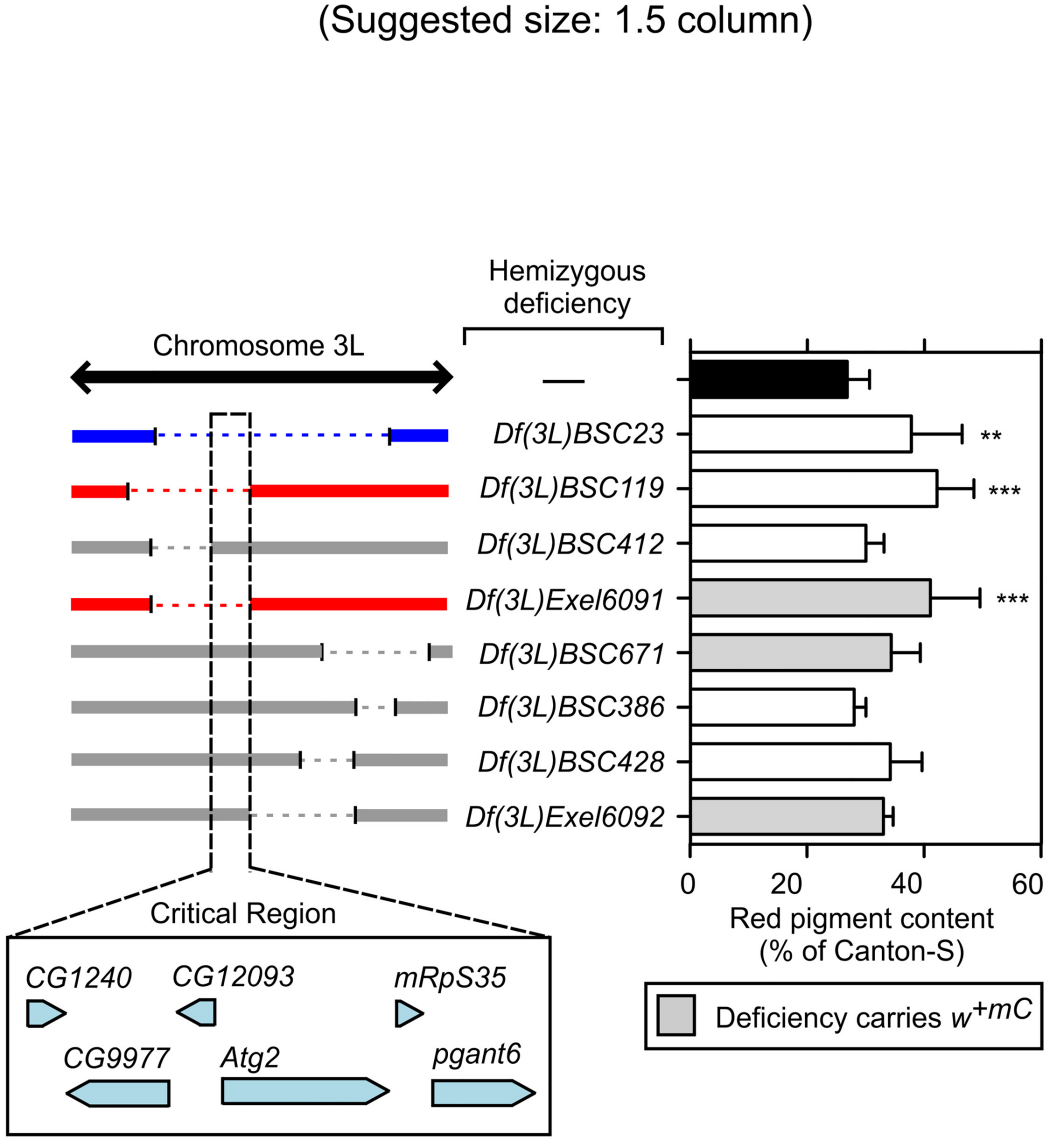
Validation and fine mapping of the critical region responsible for the modifier effect observed for *Df(3L)BSC23*. Red pigments were extracted from the heads of adult *g* ^*2*^ mutant males carrying no deletions (—) or a single copy of the indicated deficiencies, quantified as described under Materials and Methods, and expressed as percentages of the red pigment content of male flies of the wild-type (Canton-S) line. Bars represent means + SD of 3–15 biological replicates. One-way ANOVA followed by Dunnett’s test of each group versus *g* ^*2*^ flies carrying no deletion (black bar): **p<0.01, ***p<0.001. Shown on the left is a schematic representation of the extent of overlap between the chromosomal region deleted in the deficiency that had been identified through screening (blue) and those deleted in independent deficiencies that elicited (red) or failed to elicit (grey) a similar modifier effect on the *g* ^*2*^ eye color phenotype. The critical genomic region responsible for the observed modifier effect is highlighted with black dashed lines, and the relative location of the six genes found within this region (adapted from the FlyBase database) is depicted at the bottom.

In the case of *Df(3L)BSC23*, seven independent deficiencies were available to cover the entire genomic region deleted in the original deficiency; three of them carried the *w* ^*+mC*^ construct (Fig 5), with expression levels that were deemed low to moderate on the basis of pigmentation of flies carrying them on a White-null background (Fig 3A). When assessed for their ability to modify the phenotype of *g* ^*2*^ flies, only one overlapping deficiency carrying a ‘weak’ *w* ^*+mC*^ construct, *Df(3L)Exel6091*, and one deficiency lacking *w* ^*+mC*^, *Df(3L)BSC119*, elicited a partial suppressor effect similar to that observed for the original deficiency (Fig 5). Through analysis of the genomic regions deleted in these deficiencies, a critical region encompassing six annotated genes was defined (Fig 5, left bottom box).

Practical limitations precluded a systematic investigation of each of the genes included in the critical regions derived from *Df(3L)eyg* ^*C1*^ and *Df(3L)BSC23*. These limitations were: the very limited availability of loss-of-function alleles of single genes, and the inconsistency of results that we obtained using available RNAi transgenes (expressed with the aid of a *GMR-GAL4* driver [39]), with the latter likely arising from off-target effects [59]. For this reason, a candidate-gene approach was undertaken, as described in the following sections.

### *Atg2* as a genetic modifier of fly eye pigmentation

**Fig 6:**
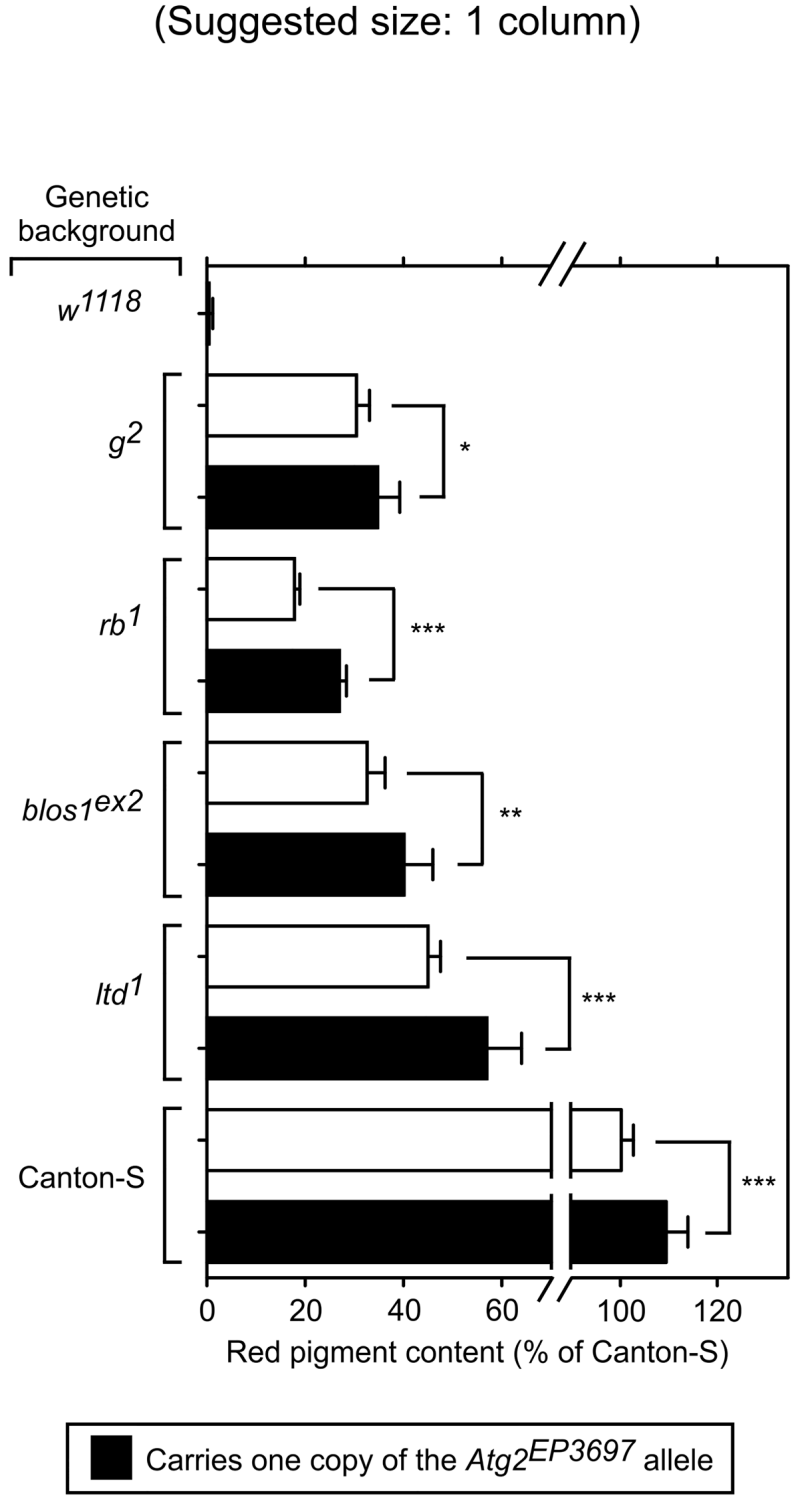
Effects of the *Atg2* ^*EP3697*^ allele on red pigment content in various genetic backgrounds. Red pigments were extracted from the heads of adult male flies of the indicated genetic backgrounds lacking (open bars) or carrying (filled bars) one copy of the *Atg2* ^*EP3697*^ allele over a normal chromosome 3. The extracted pigments were quantified as described under Materials and Methods, and the resulting values expressed as percentages of the pigment content of wild-type (Canton-S) flies. Bars represent means + SD of 7–15 biological replicates. One-way ANOVA followed by Bonferroni comparison of selected group pairs: *p<0.05, **p<0.01, ***p<0.001. Notice that a single copy of *Atg2* ^*EP3697*^ over a normal *Atg2* allele increased pigmentation of two AP-3-subunit mutants (*g* ^*2*^ and *rb* ^*1*^), a BLOC-1-subunit mutant (*blos1* ^*ex2*^), and a mutant in the Rab-GTPase Lightoid (*ltd* ^*1*^), though it also increased the red pigment content of wild-type flies. Although the transposon inserted in *Atg2* ^*EP3697*^ carries the *w* ^*+mC*^ marker, its weak activity led to barely detectable red pigments in a White-null background (*w* ^*1118*^).

An obvious candidate out of the six genes located within the critical region derived from *Df(3L)BSC23* was *Atg2*, which encodes a conserved protein involved in autophagy [60]. Genetic links between autophagy and pigmentation defects had been previously described in mammals [61] and flies [62], though none of them involved *Atg2* in particular. A loss-of-function allele of this gene, *Atg2* ^*EP3697*^, had been previously validated in assays of autophagy function in the larval fat body [63,64]. Homozygous *Atg2* ^*EP3697*^ flies die before reaching adulthood [60], whereas heterozygotes are viable with no apparent eye morphological phenotypes. Although the *Atg2* ^*EP3697*^ allele carries a copy of the *w* ^*+mC*^ construct, its expression was deemed to be quite low on the basis of the red pigment content of White-null flies carrying one copy of the allele (Fig 6).

Satisfyingly, a single copy of the *Atg2* ^*EP3697*^ allele (in heterozygous form over a normal third chromosome) elicited statistically significant increases in the eye pigment levels of the AP-3 mutants *g* ^*2*^ and *rb* ^*1*^ (Fig 6). A single copy of the same allele also increased the red pigment content of Canton-S flies (Fig 6), an effect which was in line with that originally observed for the *Df(3L)BSC23* deficiency (Table 1). In these three genetic backgrounds, the effect of the *Atg2* ^*EP3697*^ allele on eye pigmentation was less pronounced than that observed for *Df(3L)BSC23*; we speculate that either *Atg2* ^*EP3697*^ might not represent a true null allele of this gene or that other genes within the critical region might have contributed to the effect observed for the deficiency.

Next, we tested for effects of the *Atg2* ^*EP3697*^ allele on the phenotypes of another two mutants of the pigment granule group, namely *blos1* ^*ex2*^ and *ltd* ^*1*^. The *blos1* gene encodes a subunit of the fly counterpart of mammalian BLOC-1 [39,65], which like AP-3 is involved in intracellular protein trafficking and the biogenesis of LROs [22,25,26,66–68]. Although the two complexes have been shown to interact physically with each other [25–27,69–71], epistatic analyses in mice and flies have suggested that BLOC-1 and AP-3 can function, at least in part, independently of each other [26,30,39]. The *lightoid* (*ltd*) gene encodes the single fly counterpart of two closely related mammalian GTPases of the Rab family, named Rab32 and Rab38; both mammalian proteins as well as the Lightoid protein are involved in the biogenesis of LROs [37,72–74]. While mammalian Rab32 and Rab38 have been shown to interact physically and functionally with AP-3 [28], epistatic analyses have suggested that fly Lightoid and AP-3 can function, at least in part, independently of each other [37]. As shown in Fig 6, a single copy of the *Atg2* ^*EP3697*^ allele elicited small but statistically significant increases in the eye pigment contents of homozygous *blos1* ^*ex2*^ and *ltd* ^*1*^ mutants.

Together, these results suggest that the gene dosage of *Atg2* influences the eye pigmentation phenotype of AP-3 mutants as well as of other mutations affecting pigment granule biogenesis. Because the eye pigmentation of wild-type flies was likewise modified, it is tempting to speculate that the product of *Atg2* may influence pigmentation by a mechanism distinct from those mediated by AP-3, BLOC-1 and Lightoid. Thus, one may envision that autophagy–at least a form of it that it is sensitive to partial reductions in Atg2 activity–could be involved in the turnover of pigment granules. In such a scenario, decreased pigment granule degradation in *Atg2* ^*EP3697*^ heterozygotes would explain the increase in eye pigment content observed for all of the genetic backgrounds tested. Furthermore, the extent of pigment content increase could depend on how impaired autophagy of these granules might be in *Atg2* ^*EP3697*^ heterozygotes as well as on the rate of pigment granule turnover in the different genetic backgrounds (e.g., abnormal pigment granules in mutant flies might be targeted for degradation at a rate higher than that of normal granules in wild-type flies). Although the notion of organelle turnover by autophagy is widely accepted, and extensively documented for compartments such as mitochondria or peroxisomes [75], evidence for a direct role of autophagy in the degradation pigment granules (or melanosomes, in mammals) remains quite limited. For example, autophagosomes containing melanosomal proteins were observed in human melanocytes deficient in the HPS1 subunit of BLOC-3 [76]–a protein complex that functions in the biogenesis of LROs through activation of Rab32 and Rab38 [77]–and compartments described as “autophagic melano-lysosomes” were found in retinal pigment epithelium from apparently normal rhesus monkeys [78]. In addition, it has recently been reported that melanosomes transferred from melanocytes to keratinocytes in human skin can be degraded by autophagy [79]. On the other hand, published work using mammalian melanoma cell lines has uncovered a surprising link between specific components of the machinery for autophagy and the regulation of melanosome biogenesis, with some of these components promoting (e.g., the human Atg18 homologue WIPI1 [80,81]) and others antagonizing (e.g., the human Atg1 homologue ULK1 [82]) melanin accumulation in cell culture. Given this reported link between autophagy and the biogenesis of mammalian melanosomes, a putative role for Atg2 in the regulation of fly pigment granule biogenesis should be considered.

From a genetic perspective, two aspects of the identification of *Atg2* in our genetic modifier screening are worth noting. First, so far genetic defects in autophagy-related genes in humans or mice have been associated with reduced pigmentation [80,83,84], not with increased pigment content. Second, it is not clear why out of the large number of known autophagy-related genes [60] only *Atg2* was isolated in a screening designed to cover >92% of fly autosomal chromosomes (a small subset of these genes, including *Atg5* and *Atg8a*, were excluded from the screening because of their localization to fly chromosome X). Plausible explanations include genetic redundancy among groups of paralogs with partially overlapping functions, as it might be the case for the related *Atg18a* and *Atg18b* genes encoding alternative binding partners of Atg2 [64], and gene dosage sensitivity, i.e., loss of one of the two copies of a gene may not necessarily translate into significantly decreased physiological function of the gene product. These issues notwithstanding, the possibility of the Atg2 protein playing a specific function related to pigment granule dynamics (biogenesis or degradation), besides its role as a core component of the autophagy machinery [60], should deserve consideration for future studies.

### *ArfGAP1* as a dominant genetic modifier of AP-3 mutants

Two genes within the critical region inferred for the *Df(3L)eyg* ^*C1*^ deficiency (Fig 4) and with available loss-of-function alleles were considered as candidates of interest. The first candidate was *eyegone* (*eyg*), which encodes a member of the Pax6 family of transcription factors involved in retina development [85]. While the eyes of homozygous *eyg* ^*1*^ mutants are drastically reduced in size [85], heterozygous mutants display apparently normal eye morphology. In contrast with the *Df(3L)eyg* ^*C1*^ deficiency, however, a single copy of the *eyg* ^*1*^ allele failed to modify the eye pigmentation phenotype of *g* ^*2*^ mutants. Thus, the red pigment contents (expressed as percentage of wild-type levels) were 29.5 ± 2.1 for *g* ^*2*^ and 30.0 ± 3.4 for *g* ^*2*^ *;eyg* ^*1*^/+ (means ± SD; n = 6 and 9 respectively). In light of this result, this candidate was not pursued any further.

**Fig 7:**
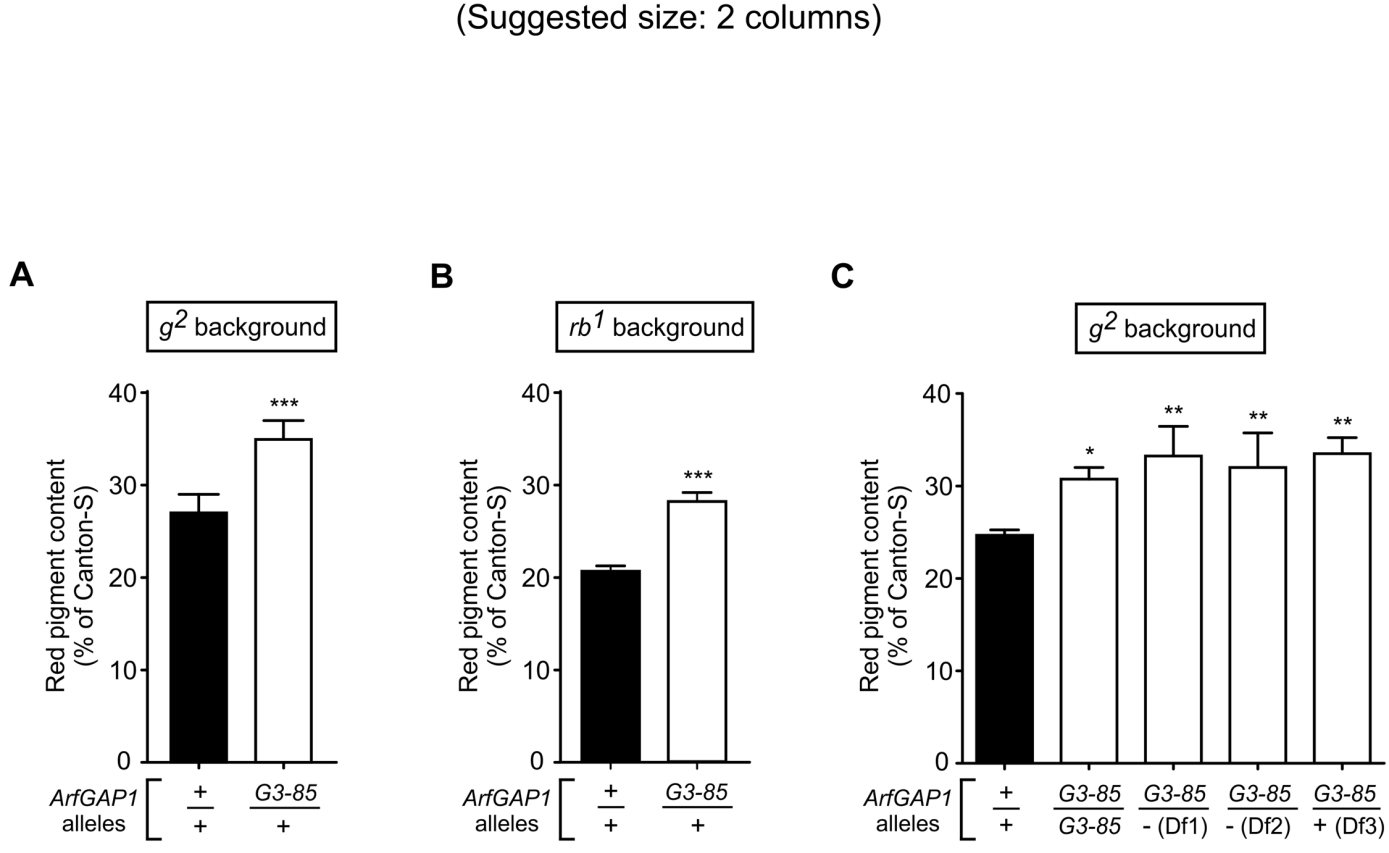
*ArfGAP1* as a modifier of the AP-3 eye pigmentation phenotype. (A-C) Red pigments were extracted from the heads of adult male flies of the indicated genetic backgrounds carrying wild-type (+) or mutant (*G3-85*) alleles of the *ArfGAP1* gene on chromosome 3. The extracted pigments were quantified as described under Materials and Methods, and the resulting values expressed as percentages of the pigment content of wild-type (Canton-S) flies. Bars represent means + SD of 3–17 biological replicates. Statistical analyses were performed by means of Student’s t-test (A and B) or one-way ANOVA followed by Dunnett’s test of each group versus *g* ^*2*^ flies carrying no deletion (C): *p<0.05, **p<0.01, ***p<0.001. Notice that a single copy of *ArfGAP1* ^*G3-85*^ mutant allele over wild-type *ArfGAP1* was sufficient to ameliorate the pigmentation defects of both *g* ^*2*^ (A) and *rb* ^*1*^ (B) AP-3-subunit mutants. Notice in (C) that such partial suppressor effect was not exacerbated in flies homozygous for the *ArfGAP1* ^*G3-85*^ allele or heterozygous for this allele over any of two deficiencies in which the deleted genomic regions include the entire *ArfGAP1* gene, namely *Df(3L)eyg* ^*C1*^ (Df1) and *Df(3L)BSC380* (Df2). The deficiency *Df(3L)BSC413*, in which the deleting genomic region excludes the *ArfGAP1* gene, was used as a control (Df3).

**Fig 8:**
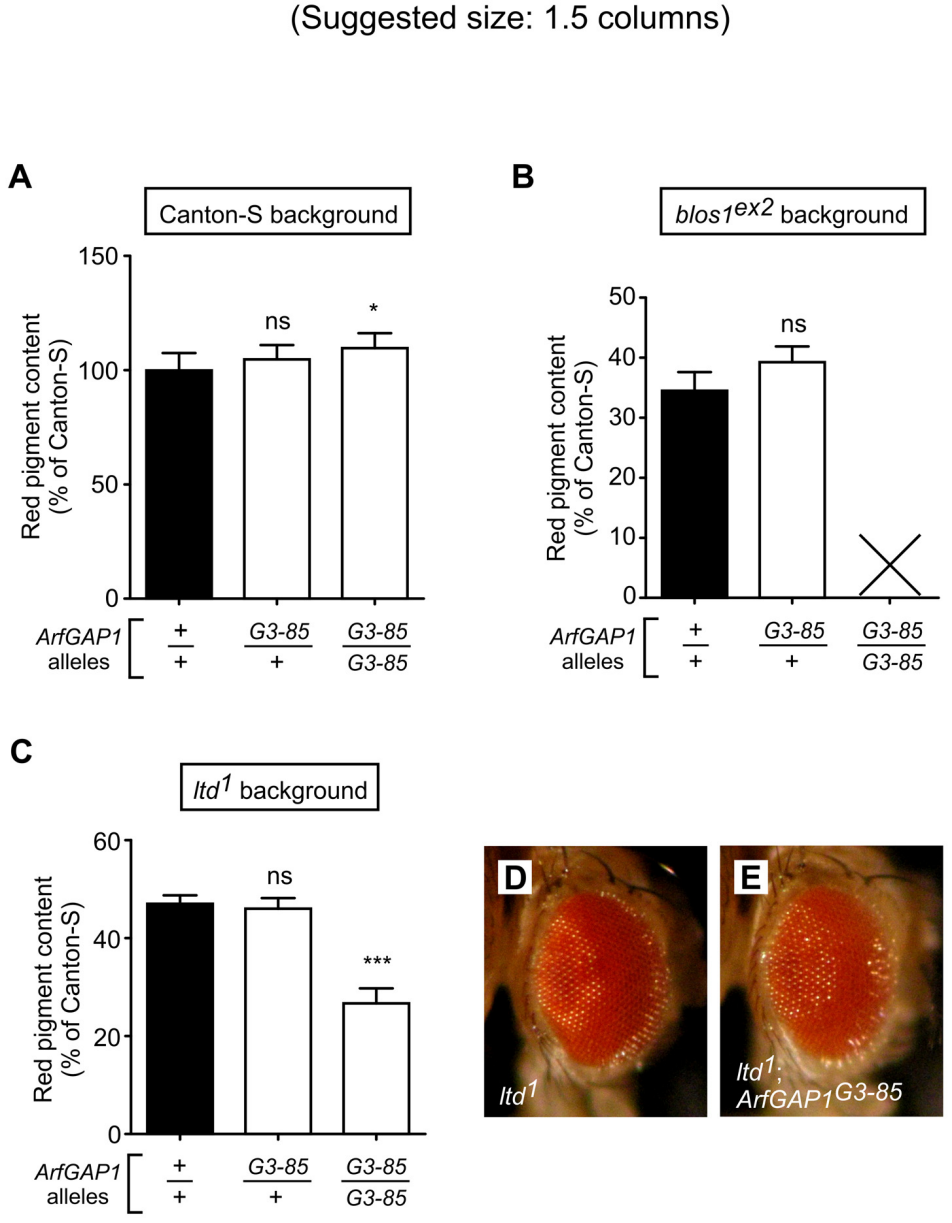
Homozygous ArfGAP1-null mutants display pleiotropic effects depending on genetic background. (A-C) Genetic crosses were set up to obtain flies carrying wild-type (+) or null mutant (*G3-85*) alleles of the *ArfGAP1* gene in the genetic backgrounds of the wild-type line Canton-S (A), the BLOC-1-subunit mutant *blos1* ^*ex2*^ (B) and the Lightoid GTPase mutant *ltd* ^*1*^ (C). Notice in (B) that males that were double homozygous for *ArfGAP1* ^*G3-85*^ and *blos1* ^*ex2*^ did not survive to adulthood (indicated with X). In those cases in which viable adult males were obtained, red pigments were extracted and quantified as described under Materials and Methods. Values were expressed as percentages of the pigment content of wild-type flies. Bars represent means + SD of 3–9 biological replicates. Statistical analyses were performed by means of Student’s t-test (B) or one-way ANOVA followed by Dunnett’s test of each group versus that of flies homozygous for the wild-type *ArfGAP1* allele in the corresponding genetic background (A and C): ns, not significant; *p<0.05, ***p<0.001. (D and E) eye morphology of adult flies homozygous for *ltd* ^*1*^ (D) and for both *ltd* ^*1*^ and *ArfGAP1* ^*G3-85*^ (E), with the latter displaying a mild interommatidial facets phenotype.

The second candidate was *ArfGAP1* (formerly known as *Gap69C*), which encodes a conserved GTPase-activating protein (GAP) with specificity to promote hydrolysis of GTP bound to members of the Arf protein family [52,86]. Flies carrying the null allele *ArfGAP1* ^*G3-85*^, either in homozygous or heterozygous form, are viable and display no eye morphological abnormalities [52]. Here, a single copy of the *ArfGAP1* ^*G3-85*^ allele elicited statistically significant increases in the pigment contents of the AP-3 mutants *g* ^*2*^ (Fig 7A) and *rb* ^*1*^ (Fig 7B). On the other hand, and consistently with the behavior observed for the *Df(3L)eyg* ^*C1*^ deficiency (Table 1), a single copy of the *ArfGAP1* ^*G3-85*^ allele failed to modify the red pigment content of wild-type flies (Fig 8A). Interestingly, the modifier effect elicited by the *ArfGAP1* ^*G3-85*^ allele in the *g* ^*2*^ background appeared to follow a dominant pattern. Thus, eye pigmentation increases similar to those observed for *g* ^*2*^ flies heterozygous for *ArfGAP1* ^*G3-85*^ over a normal third chromosome (Fig 7A) were observed not only for *g* ^*2*^ flies that were heterozygous for *ArfGAP1* ^*G3-85*^ over a control deletion (referred in the figure to as ‘Df3’) but also for those that were homozygous for the *ArfGAP1* ^*G3-85*^ mutation or compound heterozygous for *ArfGAP1* ^*G3-85*^ over either of two large deletions (‘Df1’ and ‘Df2’) encompassing the entire *ArfGAP1* gene (Fig 7C).

As described in the previous section for the *Atg2* ^*EP3697*^ mutant allele, we next tested for effects of the *ArfGAP1* ^*G3-85*^ allele on the phenotypes of *blos1* ^*ex2*^ and *ltd* ^*1*^ mutants (deficient in BLOC-1 and the Rab GTPase Lightoid, respectively). The results were remarkable. On the one hand, a single copy of *ArfGAP1* ^*G3-85*^ did not modify significantly the eye color phenotypes of homozygous *blos1* ^*ex2*^ and *ltd* ^*1*^ flies (Fig 8B and 8C), suggesting that the dominant modifier effect observed for *ArfGAP1* ^*G3-85*^ in the genetic backgrounds of *g* ^*2*^ (Fig 7A) and *rb* ^*1*^ (Fig 7B) reflected a specific functional interaction between ArfGAP1 and the AP-3 complex. On the other hand, in homozygous form the *ArfGAP1* ^*G3-85*^ allele caused drastic effects, namely: synthetic lethality of males in the background of *blos1* ^*ex2*^ homozygous (i.e., no doubly homozygous males survived to adulthood), and abnormal eye morphology (i.e., disrupted organization of interommatidial bristles) with decreased pigmentation in the background of *ltd* ^*1*^ homozygotes (Fig 8C–8E). These strong effects, which were also observed using compound heterozygous of *ArfGAP1* ^*G3-85*^ over deletions of the entire *ArfGAP1* gene [using deficiencies *Df(3L)BSC380* and *Df(3L)ED4483* for *blos1* ^*ex2*^ and the former for *ltd* ^*1*^], were at odds with those observed by *ArfGAP1* ^*G3-85*^ in homozygous form in the genetic backgrounds of *g* ^*2*^ (Fig 7C) or Canton-S (Fig 8A), where neither early lethality nor abnormal eye morphology were noted.

Taken together, the results suggest that *ArfGAP1* may be a dominant genetic modifier of AP-3 function and interact genetically with *blos1* or *ltd* in a recessive fashion. Because of the multiple cellular functions ascribed to the conserved ArfGAP1 protein in various organisms [86,87], several scenarios seem plausible. For example, given that the recruitment of the AP-3 complex to membranes is promoted by an Arf family member (possibly Arf1) in its GTP-bound form [88–91], one could envision that decreased Arf GAP activity (due to reduced dosage of the *ArfGAP1* gene) might partially compensate for incomplete impairment of AP-3 function (as in the genetic backgrounds of *g* ^*2*^ and *rb* ^*1*^) by slowing down inactivation of GTP-bound Arf molecules at the site(s) of AP-3 function. While this relatively simple scenario could explain the specificity and even the dominant pattern of the effects elicited by the *ArfGAP1* ^*G3-85*^ allele on AP-3 mutants, a few potential shortcomings should be noted. Firstly, at least in mammalian cell lines the regulation of AP-3 function through inactivation of Arf-GTP has been ascribed to a different Arf GAP, named AGAP1 [24], for which a clearly recognizable homologue (encoded by *ceng1A*) exists in flies [92]. Secondly, ArfGAP1 has well-documented activities besides inactivation of Arf GTPases, including serving as effectors of GTP-bound Arf proteins and having Arf-independent functions [86,87]. Finally, such a simple model would not provide a straightforward explanation for the strong genetic interactions observed for homozygous double mutants in *ArfGAP1* and either *blos1* (synthetic lethality in males) or *ltd* (abnormal eye morphology), as genetic interactions between AP-3 mutants and *blos1* or *ltd* alleles have been documented to affect eye pigment content but neither viability nor eye morphology [37,39]. Along these lines, one should consider that a second Arf-dependent protein trafficking pathway, defined by the structurally related AP-1 complex, has been implicated in the biogenesis of mammalian melanosomes [21,22,28,93] and might be likewise operational in the biogenesis of pigment granules in the fly eye. Accordingly, one could envision that reduced activity of ArfGAP1, which in mammals has been shown to interact physically with AP-1 [94], might exacerbate the activity of the AP-1-dependent pathway–at least in the context of impaired AP-3 function. Because AP-1, unlike AP-3, is essential for *Drosophila* larval development [95], invoking a putative role for ArfGAP1 in the AP-1-dependent pathway could provide possible explanations for the abnormalities observed in homozygous double mutants for *ArfGAP1* and *blos1* as well as for *ArfGAP1* and *ltd*. These considerations notwithstanding, a scenario whereby ArfGAP1 would act on an AP-1-dependent pathway would not represent the only plausible alternative to that of a direct role in AP-3-dependent events. For example, recent studies have demonstrated that ArfGAP1 from mammals and flies can interact physically and functionally with a leucine-rich repeat kinase, named LRRK2 in mammals and Lrrk in flies [96,97], which in turn has been shown to function in the endosomal-lysosomal system and autophagy of both flies and mammals [98–101] in part through interactions with Rab GTPases such as Rab7 [100] and–most notably–Lightoid and its mammalian counterparts [102,103]. Accordingly, decreased ArfGAP1 activity could have elicited the modifier effects reported in this paper through alteration of the function of proteins such as Lrrk. Future research will be required to test experimentally the validity of these and other plausible scenarios.

## Conclusions

Our genetic modifier screening has led to the identification of distinct regions within fly chromosome 3, and in particular of two candidate genes, which upon deletion or mutation in heterozygous form modified the eye pigmentation phenotype of AP-3 mutants. One of these candidate genes encodes the single fly counterpart of Atg2, a conserved component of the molecular machinery for autophagy. The other gene encodes the single fly counterpart of ArfGAP1, a conserved protein implicated in Arf-dependent protein trafficking events as well as in Lrrk-dependent regulation of the endosomal-lysosomal system and autophagy.

## References

[1] Dell'Angelica EC (2009) AP-3-dependent trafficking and disease: the first decade. Curr Opin Cell Biol 21: 552–559. 10.1016/j.ceb.2009.04.014 19497727

[2] Hirst J, Barlow LD, Francisco GC, Sahlender DA, Seaman MN, Dacks JB, et al (2012) The fifth adaptor protein complex. PLoS Biol 9: e1001170. 10.1371/journal.pbio.1001170 PMC3191125 22022230

[3] Canagarajah BJ, Ren X, Bonifacino JS, Hurley JH (2013) The clathrin adaptor complexes as a paradigm for membrane-associated allostery. Protein Sci 22: 517–529. 10.1002/pro.2235 23424177 PMC3649254

[4] Dell'Angelica EC, Shotelersuk V, Aguilar RC, Gahl WA, Bonifacino JS (1999) Altered trafficking of lysosomal proteins in Hermansky-Pudlak syndrome due to mutations in the β3A subunit of the AP-3 adaptor. Mol Cell 3: 11–21. 10024875 10.1016/s1097-2765(00)80170-7

[5] Huizing M, Scher CD, Strovel E, Fitzpatrick DL, Hartnell LM, Anikster Y, et al (2002) Nonsense mutations in ADTB3A cause complete deficiency of the β3A subunit of adaptor complex-3 and severe Hermansky-Pudlak syndrome type 2. Pediatr Res 51: 150–158. 11809908 10.1203/00006450-200202000-00006

[6] Fontana S, Parolini S, Vermi W, Booth S, Gallo F, Donini M, et al (2006) Innate immunity defects in Hermansky-Pudlak type 2 syndrome. Blood 107: 4857–4864. 16507770 10.1182/blood-2005-11-4398

[7] Jung J, Bohn G, Allroth A, Boztug K, Brandes G, Sandrock I, et al (2006) Identification of a homozygous deletion in the AP3B1 gene causing Hermansky-Pudlak syndrome, type 2. Blood 108: 362–369. 16537806 10.1182/blood-2005-11-4377 PMC1895843

[8] Enders A, Zieger B, Schwarz K, Yoshimi A, Speckmann C, Knoepfle EM, et al (2006) Lethal hemophagocytic lymphohistiocytosis in Hermansky-Pudlak syndrome type II. Blood 108: 81–87. 16551969 10.1182/blood-2005-11-4413

[9] Wenham M, Grieve S, Cummins M, Jones ML, Booth S, Kilner R, et al (2010) Two patients with Hermansky Pudlak syndrome type 2 and novel mutations in AP3B1. Haematologica 95: 333–337. 10.3324/haematol.2009.012286 19679886 PMC2817039

[10] Kurnik K, Bartsch I, Maul-Pavicic A, Ehl S, Sandrock-Lang K, Bidlingmaier C, et al (2013) Novel mutation in Hermansky-Pudlak syndrome type 2 with mild immunological phenotype. Platelets 24: 538–543. 10.3109/09537104.2012.741275 23215637

[11] Jones ML, Murden SL, Brooks C, Maloney V, Manning RA, Gilmour KC, et al (2013) Disruption of AP3B1 by a chromosome 5 inversion: a new disease mechanism in Hermansky-Pudlak syndrome type 2. BMC Med Genet 14: 42 10.1186/1471-2350-14-42 23557002 PMC3663694

[12] Huizing M, Helip-Wooley A, Westbroek W, Gunay-Aygun M, Gahl WA (2008) Disorders of lysosome-related organelle biogenesis: clinical and molecular genetics. Annu Rev Genomics Hum Genet 9: 359–386. 10.1146/annurev.genom.9.081307.164303 18544035 PMC2755194

[13] Bonifacino JS, Traub LM (2003) Signals for sorting of transmembrane proteins to endosomes and lysosomes. Annu Rev Biochem 72: 395–447. 12651740 10.1146/annurev.biochem.72.121801.161800

[14] Robinson MS (2004) Adaptable adaptors for coated vesicles. Trends Cell Biol 14: 167–174. 15066634 10.1016/j.tcb.2004.02.002

[15] Newell-Litwa K, Seong E, Burmeister M, Faundez V (2007) Neuronal and non-neuronal functions of the AP-3 sorting machinery. J Cell Sci 120: 531–541. 17287392 10.1242/jcs.03365

[16] Le Borgne R, Alconada A, Bauer U, Hoflack B (1998) The mammalian AP-3 adaptor-like complex mediates the intracellular transport of lysosomal membrane glycoproteins. J Biol Chem 273: 29451–29461. 9792650 10.1074/jbc.273.45.29451

[17] Yang W, Li C, Ward DM, Kaplan J, Mansour SL (2000) Defective organellar membrane protein trafficking in Ap3b1-deficient cells. J Cell Sci 113: 4077–4086. 11058094 10.1242/jcs.113.22.4077

[18] Peden AA, Rudge RE, Lui WW, Robinson MS (2002) Assembly and function of AP-3 complexes in cells expressing mutant subunits. J Cell Biol 156: 327–336. 11807095 10.1083/jcb.200107140 PMC2199225

[19] Peden AA, Oorschot V, Hesser BA, Austin CD, Scheller RH, Klumperman J (2004) Localization of the AP-3 adaptor complex defines a novel endosomal exit site for lysosomal membrane proteins. J Cell Biol 164: 1065–1076. 15051738 10.1083/jcb.200311064 PMC2172074

[20] Huizing M, Sarangarajan R, Strovel E, Zhao Y, Gahl WA, Boissy RE (2001) AP-3 mediates tyrosinase but not TRP-1 trafficking in human melanocytes. Mol Biol Cell 12: 2075–2085. 11452004 10.1091/mbc.12.7.2075 PMC55657

[21] Theos AC, Tenza D, Martina JA, Hurbain I, Peden AA, Sviderskaya EV, et al (2005) Functions of adaptor protein (AP)-3 and AP-1 in tyrosinase sorting from endosomes to melanosomes. Mol Biol Cell 16: 5356–5372. 16162817 10.1091/mbc.E05-07-0626 PMC1266432

[22] Sitaram A, Dennis MK, Chaudhuri R, De Jesus-Rojas W, Tenza D, Setty SR, et al (2012) Differential recognition of a dileucine-based sorting signal by AP-1 and AP-3 reveals a requirement for both BLOC-1 and AP-3 in delivery of OCA2 to melanosomes. Mol Biol Cell 23: 3178–3192. 22718909 10.1091/mbc.E11-06-0509 PMC3418312

[23] Dell'Angelica EC, Klumperman J, Stoorvogel W, Bonifacino JS (1998) Association of the AP-3 adaptor complex with clathrin. Science 280: 431–434. 9545220 10.1126/science.280.5362.431

[24] Nie Z, Boehm M, Boja ES, Vass WC, Bonifacino JS, Fales HM, et al (2003) Specific regulation of the adaptor protein complex AP-3 by the Arf GAP AGAP1. Dev Cell 5: 513–521. 12967569 10.1016/s1534-5807(03)00234-x

[25] Salazar G, Craige B, Styers ML, Newell-Litwa KA, Doucette MM, Wainer BH, et al (2006) BLOC-1 complex deficiency alters the targeting of adaptor protein complex-3 cargoes. Mol Biol Cell 17: 4014–4026. 16760431 10.1091/mbc.E06-02-0103 PMC1556383

[26] Di Pietro SM, Falcon-Perez JM, Tenza D, Setty SR, Marks MS, Raposo G, et al (2006) BLOC-1 interacts with BLOC-2 and the AP-3 complex to facilitate protein trafficking on endosomes. Mol Biol Cell 17: 4027–4038. 16837549 10.1091/mbc.E06-05-0379 PMC1593172

[27] Salazar G, Zlatic S, Craige B, Peden AA, Pohl J, Faundez V (2009) Hermansky-Pudlak syndrome protein complexes associate with phosphatidylinositol 4-kinase type II α in neuronal and non-neuronal cells. J Biol Chem 284: 1790–1802. 10.1074/jbc.M805991200 19010779 PMC2615517

[28] Bultema JJ, Ambrosio AL, Burek CL, Di Pietro SM (2012) BLOC-2, AP-3, and AP-1 proteins function in concert with Rab38 and Rab32 proteins to mediate protein trafficking to lysosome-related organelles. J Biol Chem 287: 19550–19563. 10.1074/jbc.M112.351908 22511774 PMC3365991

[29] Feng L, Novak EK, Hartnell LM, Bonifacino JS, Collinson LM, Swank RT (2002) The Hermansky-Pudlak syndrome 1 (HPS1) and HPS2 genes independently contribute to the production and function of platelet dense granules, melanosomes, and lysosomes. Blood 99: 1651–1658. 11861280

[30] Gautam R, Novak EK, Tan J, Wakamatsu K, Ito S, Swank RT (2006) Interaction of Hermansky-Pudlak Syndrome genes in the regulation of lysosome-related organelles. Traffic 7: 779–792. 16787394 10.1111/j.1600-0854.2006.00431.x

[31] Hoyle DJ, Rodriguez-Fernandez IA, Dell'angelica EC (2011) Functional interactions between OCA2 and the protein complexes BLOC-1, BLOC-2, and AP-3 inferred from epistatic analyses of mouse coat pigmentation. Pigment Cell Melanoma Res 24: 275–281. 10.1111/j.1755-148X.2010.00815.x 21392365 PMC3070960

[32] Rinchik EM, Bultman SJ, Horsthemke B, Lee ST, Strunk KM, Spritz RA, et al (1993) A gene for the mouse pink-eyed dilution locus and for human type II oculocutaneous albinism. Nature 361: 72–76. 8421497 10.1038/361072a0

[33] Schroeder LK, Kremer S, Kramer MJ, Currie E, Kwan E, Watts JL, et al (2007) Function of the Caenorhabditis elegans ABC transporter PGP-2 in the biogenesis of a lysosome-related fat storage organelle. Mol Biol Cell 18: 995–1008. 17202409 10.1091/mbc.E06-08-0685 PMC1805080

[34] Rabbitts BM, Ciotti MK, Miller NE, Kramer M, Lawrenson AL, Levitte S, et al (2008) glo-3, a novel Caenorhabditis elegans gene, is required for lysosome-related organelle biogenesis. Genetics 180: 857–871. 10.1534/genetics.108.093534 18780725 PMC2567386

[35] Hermann GJ, Scavarda E, Weis AM, Saxton DS, Thomas LL, Salesky R, et al (2012) C. elegans BLOC-1 functions in trafficking to lysosome-related gut granules. PLoS One 7: e43043 10.1371/journal.pone.0043043 22916203 PMC3419718

[36] Delahaye JL, Foster OK, Vine A, Saxton DS, Curtin TP, Somhegyi H, et al (2014) Caenorhabditis elegans HOPS and CCZ-1 mediate trafficking to lysosome-related organelles independently of RAB-7 and SAND-1. Mol Biol Cell 25: 1073–1096. 10.1091/mbc.E13-09-0521 24501423 PMC3967972

[37] Ma J, Plesken H, Treisman JE, Edelman-Novemsky I, Ren M (2004) Lightoid and Claret: a rab GTPase and its putative guanine nucleotide exchange factor in biogenesis of Drosophila eye pigment granules. Proc Natl Acad Sci U S A 101: 11652–11657. 15289618 10.1073/pnas.0401926101 PMC511034

[38] Falcon-Perez JM, Romero-Calderon R, Brooks ES, Krantz DE, Dell'Angelica EC (2007) The Drosophila pigmentation gene pink (p) encodes a homologue of human Hermansky-Pudlak syndrome 5 (HPS5). Traffic 8: 154–168. 17156100 10.1111/j.1600-0854.2006.00514.x

[39] Cheli VT, Daniels RW, Godoy R, Hoyle DJ, Kandachar V, Starcevic M, et al (2010) Genetic modifiers of abnormal organelle biogenesis in a Drosophila model of BLOC-1 deficiency. Hum Mol Genet 19: 861–878. 10.1093/hmg/ddp555 20015953 PMC2816613

[40] Holmes A, Flett A, Coudreuse D, Korswagen HC, Pettitt J (2007) C. elegans Disabled is required for cell-type specific endocytosis and is essential in animals lacking the AP-3 adaptor complex. J Cell Sci 120: 2741–2751. 17636000 10.1242/jcs.03474

[41] Lloyd VK, Sinclair DA, Alperyn M, Grigliatti TA (2002) Enhancer of garnet/δAP-3 is a cryptic allele of the white gene and identifies the intracellular transport system for the white protein. Genome 45: 296–312. 11962627 10.1139/g01-139

[42] Simonsen A, Cumming RC, Lindmo K, Galaviz V, Cheng S, Rusten TE, et al (2007) Genetic modifiers of the Drosophila blue cheese gene link defects in lysosomal transport with decreased life span and altered ubiquitinated-protein profiles. Genetics 176: 1283–1297. 17435236 10.1534/genetics.106.065011 PMC1894590

[43] Finley KD, Edeen PT, Cumming RC, Mardahl-Dumesnil MD, Taylor BJ, Rodriguez MH, et al (2003) blue cheese mutations define a novel, conserved gene involved in progressive neural degeneration. J Neurosci 23: 1254–1264. 12598614 10.1523/JNEUROSCI.23-04-01254.2003 PMC1975817

[44] Wilkin M, Tongngok P, Gensch N, Clemence S, Motoki M, Yamada K, et al (2008) Drosophila HOPS and AP-3 complex genes are required for a Deltex-regulated activation of notch in the endosomal trafficking pathway. Dev Cell 15: 762–772. 10.1016/j.devcel.2008.09.002 19000840

[45] Cao W, Song HJ, Gangi T, Kelkar A, Antani I, Garza D, et al (2008) Identification of novel genes that modify phenotypes induced by Alzheimer's beta-amyloid overexpression in Drosophila. Genetics 178: 1457–1471. 10.1534/genetics.107.078394 18245849 PMC2278065

[46] Kantheti P, Qiao X, Diaz ME, Peden AA, Meyer GE, Carskadon SL, et al (1998) Mutation in AP-3 δ in the mocha mouse links endosomal transport to storage deficiency in platelets, melanosomes, and synaptic vesicles. Neuron 21: 111–122. 9697856 10.1016/s0896-6273(00)80519-x

[47] Feng L, Seymour AB, Jiang S, To A, Peden AA, Novak EK, et al (1999) The β3A subunit gene (Ap3b1) of the AP-3 adaptor complex is altered in the mouse hypopigmentation mutant pearl, a model for Hermansky-Pudlak syndrome and night blindness. Hum Mol Genet 8: 323–330. 9931340 10.1093/hmg/8.2.323

[48] Simpson F, Peden AA, Christopoulou L, Robinson MS (1997) Characterization of the adaptor-related protein complex, AP-3. J Cell Biol 19; 137: 835–845. 9151686 10.1083/jcb.137.4.835 PMC2139840

[49] Ooi CE, Moreira JE, Dell'Angelica EC, Poy G, Wassarman DA, Bonifacino JS (1997) Altered expression of a novel adaptin leads to defective pigment granule biogenesis in the Drosophila eye color mutant garnet. EMBO J 16: 4508–4518. 9303295 10.1093/emboj/16.15.4508 PMC1170077

[50] Lloyd V, Ramaswami M, Kramer H (1998) Not just pretty eyes: Drosophila eye-colour mutations and lysosomal delivery. Trends Cell Biol 8: 257–259. 9714595 10.1016/s0962-8924(98)01270-7

[51] Greenspan RJ (1997) Fly pushing: the theory and practice of Drosophila genetics New York: Cold Spring Harbor Laboratory Press.

[52] Frolov MV, Alatortsev VE (2001) Molecular analysis of novel Drosophila gene, Gap69C, encoding a homolog of ADP-ribosylation factor GTPase-activating protein. DNA Cell Biol 20: 107–113. 11244568 10.1089/104454901750070319

[53] Cook RK, Christensen SJ, Deal JA, Coburn RA, Deal ME, Gresens JM, et al (2012) The generation of chromosomal deletions to provide extensive coverage and subdivision of the Drosophila melanogaster genome. Genome Biol 13: R21 10.1186/gb-2012-13-3-r21 22445104 PMC3439972

[54] Lloyd VK, Sinclair DA, Wennberg R, Warner TS, Honda BM, Grigliatti TA (1999) A genetic and molecular characterization of the garnet gene of Drosophila melanogaster. Genome 42: 1183–1193. 10659786

[55] Kretzschmar D, Poeck B, Roth H, Ernst R, Keller A, Porsch M, et al (2000) Defective pigment granule biogenesis and aberrant behavior caused by mutations in the Drosophila AP-3β adaptin gene ruby. Genetics 155: 213–223. 10790396 10.1093/genetics/155.1.213 PMC1461058

[56] Mullins C, Hartnell LM, Bonifacino JS (2000) Distinct requirements for the AP-3 adaptor complex in pigment granule and synaptic vesicle biogenesis in Drosophila melanogaster. Mol Gen Genet 263: 1003–1014. 10954086 10.1007/pl00008688

[57] Silicheva M, Golovnin A, Pomerantseva E, Parshikov A, Georgiev P, Maksimenko O (2010) Drosophila mini-white model system: new insights into positive position effects and the role of transcriptional terminators and gypsy insulator in transgene shielding. Nucleic Acids Res 38: 39–47. 10.1093/nar/gkp877 19854952 PMC2800232

[58] Ishikawa HO, Xu A, Ogura E, Manning G, Irvine KD (2012) The Raine syndrome protein FAM20C is a Golgi kinase that phosphorylates bio-mineralization proteins. PLoS One 7: e42988 10.1371/journal.pone.0042988 22900076 PMC3416761

[59] Ma Y, Creanga A, Lum L, Beachy PA (2006) Prevalence of off-target effects in Drosophila RNA interference screens. Nature 443: 359–363. 16964239 10.1038/nature05179

[60] Mulakkal NC, Nagy P, Takats S, Tusco R, Juhász G, Nezis IP (2014) Autophagy in Drosophila: from historical studies to current knowledge. Biomed Res Int 2014: 273473 10.1155/2014/273473 24949430 PMC4052151

[61] Ho H, Ganesan AK (2011) The pleiotropic roles of autophagy regulators in melanogenesis. Pigment Cell Melanoma Res 24: 595–604. 10.1111/j.1755-148X.2011.00889.x 21777401

[62] Wang C, Liu Z, Huang X (2012) Rab32 is important for autophagy and lipid storage in Drosophila. PLoS One 7: e32086 10.1371/journal.pone.0032086 22348149 PMC3279429

[63] Scott RC, Schuldiner O, Neufeld TP (2004) Role and regulation of starvation-induced autophagy in the Drosophila fat body. Dev Cell 7: 167–178. 15296714 10.1016/j.devcel.2004.07.009

[64] Nagy P, Hegedűs K, Pircs K, Varga Á, Juhász G (2014) Different effects of Atg2 and Atg18 mutations on Atg8a and Atg9 trafficking during starvation in Drosophila. FEBS Lett 588: 408–413. 10.1016/j.febslet.2013.12.012 24374083 PMC3928829

[65] Mullin AP, Sadanandappa MK, Ma W, Dickman DK, VijayRaghavan K, Ramaswami M, et al (2015) Gene dosage in the dysbindin schizophrenia susceptibility network differentially affect synaptic function and plasticity. J Neurosci 35: 325–338. 10.1523/JNEUROSCI.3542-14.2015 25568125 PMC4287151

[66] Falcón-Pérez JM, Starcevic M, Gautam R, Dell'Angelica EC (2002) BLOC-1, a novel complex containing the pallidin and muted proteins involved in the biogenesis of melanosomes and platelet-dense granules. J Biol Chem 277: 28191–28199. 12019270 10.1074/jbc.M204011200

[67] Moriyama K, Bonifacino JS (2002) Pallidin is a component of a multi-protein complex involved in the biogenesis of lysosome-related organelles. Traffic 3: 666–677. 12191018 10.1034/j.1600-0854.2002.30908.x

[68] Setty SR, Tenza D, Truschel ST, Chou E, Sviderskaya EV, Theos AC, et al (2007) BLOC-1 is required for cargo-specific sorting from vacuolar early endosomes toward lysosome-related organelles. Mol Biol Cell 18: 768–780. 17182842 10.1091/mbc.E06-12-1066 PMC1805088

[69] Hikita T, Taya S, Fujino Y, Taneichi-Kuroda S, Ohta K, Tsuboi D, et al (2009) Proteomic analysis reveals novel binding partners of dysbindin, a schizophrenia-related protein. J Neurochem 110: 1567–1574. 10.1111/j.1471-4159.2009.06257.x 19573021

[70] Mead CL, Kuzyk MA, Moradian A, Wilson GM, Holt RA, Morin GB (2010) Cytosolic protein interactions of the schizophrenia susceptibility gene dysbindin. J Neurochem 113: 1491–1503. 10.1111/j.1471-4159.2010.06690.x 20236384

[71] Gokhale A, Larimore J, Werner E, So L, Moreno-De-Luca A, Lese-Martin C, et al (2012) Quantitative proteomic and genetic analyses of the schizophrenia susceptibility factor dysbindin identify novel roles of the biogenesis of lysosome-related organelles complex 1. J Neurosci 32: 3697–3711. 10.1523/JNEUROSCI.5640-11.2012 22423091 PMC3313842

[72] Loftus SK, Larson DM, Baxter LL, Antonellis A, Chen Y, Wu X, et al (2002) Mutation of melanosome protein RAB38 in chocolate mice. Proc Natl Acad Sci U S A 99: 4471–4476. 11917121 10.1073/pnas.072087599 PMC123672

[73] Oiso N, Riddle SR, Serikawa T, Kuramoto T, Spritz RA (2004) The rat Ruby (R) locus is Rab38: identical mutations in Fawn-hooded and Tester-Moriyama rats derived from an ancestral Long Evans rat sub-strain. Mamm Genome 15: 307–314. 15112108 10.1007/s00335-004-2337-9

[74] Wasmeier C, Romao M, Plowright L, Bennett DC, Raposo G, Seabra MC (2006) Rab38 and Rab32 control post-Golgi trafficking of melanogenic enzymes. J Cell Biol 175: 271–281. 17043139 10.1083/jcb.200606050 PMC2064568

[75] Okamoto K (2014) Organellophagy: eliminating cellular building blocks via selective autophagy. J Cell Biol 205: 435–445. 10.1083/jcb.201402054 24862571 PMC4033777

[76] Smith JW, Koshoffer A, Morris RE, Boissy RE (2005) Membranous complexes characteristic of melanocytes derived from patients with Hermansky-Pudlak syndrome type 1 are macroautophagosomal entities of the lysosomal compartment. Pigment Cell Res 18: 417–426. 16280007 10.1111/j.1600-0749.2005.00265.x PMC1635962

[77] Gerondopoulos A, Langemeyer L, Liang JR, Linford A, Barr FA (2012) BLOC-3 mutated in Hermansky-Pudlak syndrome is a Rab32/38 guanine nucleotide exchange factor. Curr Biol 22: 2135–2139. 10.1016/j.cub.2012.09.020 23084991 PMC3502862

[78] Gouras P, Brown K, Ivert L, Neuringer M (2011) A novel melano-lysosome in the retinal epithelium of rhesus monkeys. Exp Eye Res 93: 937–946. 10.1016/j.exer.2011.10.011 22056912 PMC6314486

[79] Murase D, Hachiya A, Takano K, Hicks R, Visscher MO, Kitahara T, et al (2013) Autophagy has a significant role in determining skin color by regulating melanosome degradation in keratinocytes. J Invest Dermatol 133: 2416–2424. 10.1038/jid.2013.165 23558403

[80] Ganesan AK, Ho H, Bodemann B, Petersen S, Aruri J, Koshy S, et al (2008) Genome-wide siRNA-based functional genomics of pigmentation identifies novel genes and pathways that impact melanogenesis in human cells. PLoS Genet 4: e1000298 10.1371/journal.pgen.1000298 19057677 PMC2585813

[81] Ho H, Kapadia R, Al-Tahan S, Ahmad S, Ganesan AK (2011) WIPI1 coordinates melanogenic gene transcription and melanosome formation via TORC1 inhibition. J Biol Chem 286: 12509–12523. 10.1074/jbc.M110.200543 21317285 PMC3069453

[82] Kalie E, Razi M, Tooze SA (2013) ULK1 regulates melanin levels in MNT-1 cells independently of mTORC1. PLoS One 8: e75313 10.1371/journal.pone.0075313 24066173 PMC3774811

[83] Cullup T, Kho AL, Dionisi-Vici C, Brandmeier B, Smith F, Urry Z, et al (2013) Recessive mutations in EPG5 cause Vici syndrome, a multisystem disorder with defective autophagy. Nat Genet 45: 83–87. 10.1038/ng.2497 23222957 PMC4012842

[84] Zhang CF, Gruber F, Ni C, Mildner M, Koenig U, Karner S, et al (2015) Suppression of Autophagy Dysregulates the Antioxidant Response and Causes Premature Senescence of Melanocytes. J Invest Dermatol 135: 1348–1357. 10.1038/jid.2014.439 25290687

[85] Jang CC, Chao JL, Jones N, Yao LC, Bessarab DA, Kuo YM, et al (2003) Two Pax genes, eye gone and eyeless, act cooperatively in promoting Drosophila eye development. Development 130: 2939–2951. 12756177 10.1242/dev.00522

[86] East MP, Kahn RA (2011) Models for the functions of Arf GAPs. Semin Cell Dev Biol 22: 3–9. 10.1016/j.semcdb.2010.07.002 20637885 PMC2976832

[87] Inoue H, Randazzo PA (2007) Arf GAPs and their interacting proteins. Traffic 8: 1465–1475. 17666108 10.1111/j.1600-0854.2007.00624.x

[88] Ooi CE, Dell'Angelica EC, Bonifacino JS (1998) ADP-Ribosylation factor 1 (ARF1) regulates recruitment of the AP-3 adaptor complex to membranes. J Cell Biol 142: 391–402. 9679139 10.1083/jcb.142.2.391 PMC2133064

[89] Faúndez V, Horng JT, Kelly RB (1998) A function for the AP3 coat complex in synaptic vesicle formation from endosomes. Cell 93: 423–432. 9590176 10.1016/s0092-8674(00)81170-8

[90] Drake MT, Zhu Y, Kornfeld S (2000) The assembly of AP-3 adaptor complex-containing clathrin-coated vesicles on synthetic liposomes. Mol Biol Cell 11: 3723–3736. 11071902 10.1091/mbc.11.11.3723 PMC15032

[91] Austin C, Boehm M, Tooze SA (2002) Site-specific cross-linking reveals a differential direct interaction of class 1, 2, and 3 ADP-ribosylation factors with adaptor protein complexes 1 and 3. Biochemistry 41: 4669–4677. 11926829 10.1021/bi016064j

[92] Bernards A (2003) GAPs galore! A survey of putative Ras superfamily GTPase activating proteins in man and Drosophila. Biochim Biophys Acta 1603: 47–82. 12618308 10.1016/s0304-419x(02)00082-3

[93] Delevoye C, Hurbain I, Tenza D, Sibarita JB, Uzan-Gafsou S, Ohno H, et al (2009) AP-1 and KIF13A coordinate endosomal sorting and positioning during melanosome biogenesis. J Cell Biol 187: 247–264. 10.1083/jcb.200907122 19841138 PMC2768840

[94] Rawet M, Levi-Tal S, Szafer-Glusman E, Parnis A, Cassel D (2010) ArfGAP1 interacts with coat proteins through tryptophan-based motifs. Biochem Biophys Res Commun 394: 553–557. 10.1016/j.bbrc.2010.03.017 20211604

[95] Benhra N, Lallet S, Cotton M, Le Bras S, Dussert A, Le Borgne R (2011) AP-1 controls the trafficking of Notch and Sanpodo toward E-cadherin junctions in sensory organ precursors. Curr Biol 21: 87–95. 10.1016/j.cub.2010.12.010 21194948

[96] Xiong Y, Yuan C, Chen R, Dawson TM, Dawson VL (2012) ArfGAP1 is a GTPase activating protein for LRRK2: reciprocal regulation of ArfGAP1 by LRRK2. J Neurosci 32: 3877–3886. 10.1523/JNEUROSCI.4566-11.2012 22423108 PMC3319331

[97] Stafa K, Trancikova A, Webber PJ, Glauser L, West AB, Moore DJ (2012) GTPase activity and neuronal toxicity of Parkinson's disease-associated LRRK2 is regulated by ArfGAP1. PLoS Genet 8: e1002526 10.1371/journal.pgen.1002526 22363216 PMC3280333

[98] Tong Y, Yamaguchi H, Giaime E, Boyle S, Kopan R, Kelleher RJ 3rd, et al (2010) Loss of leucine-rich repeat kinase 2 causes impairment of protein degradation pathways, accumulation of alpha-synuclein, and apoptotic cell death in aged mice. Proc Natl Acad Sci U S A 107: 9879–9884. 10.1073/pnas.1004676107 20457918 PMC2906862

[99] Gómez-Suaga P, Luzón-Toro B, Churamani D, Zhang L, Bloor-Young D, Patel S, et al (2012) Leucine-rich repeat kinase 2 regulates autophagy through a calcium-dependent pathway involving NAADP. Hum Mol Genet 21: 511–525. 10.1093/hmg/ddr481 22012985 PMC3259011

[100] Dodson MW, Zhang T, Jiang C, Chen S, Guo M (2012) Roles of the Drosophila LRRK2 homolog in Rab7-dependent lysosomal positioning. Hum Mol Genet 21: 1350–1363. 10.1093/hmg/ddr573 22171073 PMC3284123

[101] Dodson MW, Leung LK, Lone M, Lizzio MA, Guo M (2014) Novel ethyl methanesulfonate (EMS)-induced null alleles of the Drosophila homolog of LRRK2 reveal a crucial role in endolysosomal functions and autophagy in vivo. Dis Model Mech 7: 1351–1363. 10.1242/dmm.017020 25288684 PMC4257004

[102] MacLeod DA, Rhinn H, Kuwahara T, Zolin A, Di Paolo G, McCabe BD, et al (2013) RAB7L1 interacts with LRRK2 to modify intraneuronal protein sorting and Parkinson's disease risk. Neuron 77: 425–439. 10.1016/j.neuron.2012.11.033 23395371 PMC3646583

[103] Waschbüsch D, Michels H, Strassheim S, Ossendorf E, Kessler D, Gloeckner CJ, et al (2014) LRRK2 transport is regulated by its novel interacting partner Rab32. PLoS One 9: e111632 10.1371/journal.pone.0111632 25360523 PMC4216093

